# No Superior Bone Union Outcomes with Allografts Compared to No Grafts and Autografts Following Medial Opening Wedge High Tibial Osteotomy: A Retrospective Cohort Study

**DOI:** 10.1111/os.13961

**Published:** 2023-12-18

**Authors:** Yunhe Mao, Lei Yao, Junqiao Li, Jian Li, Yan Xiong

**Affiliations:** ^1^ Department of Orthopedics, Orthopedic Research Institute, West China Hospital Sichuan University Chengdu China; ^2^ Department of Orthopedics, Sports Medicine Center, West China Hospital Sichuan University Chengdu China

**Keywords:** Bone union, High tibial osteotomy, Knee osteoarthritis, Osteotomy filler

## Abstract

**Objective:**

There has been long‐standing debate about whether a medial opening wedge high tibial osteotomy (MOWHTO) gap should be filled with autologous bone graft or any other filler to expedite the healing process. The main purpose of this study was to compare the clinical and radiological outcomes of MOWHTO with an opening gap ≥10 mm, utilizing autograft, allograft, or no graft at 1 year postoperatively.

**Methods:**

A total of 68 patients were included in this retrospective study and divided into three treatment groups: Group A (no bone graft), Group B (autologous iliac crest graft), and Group C (allogenous tibia plateau graft). At postoperative 1‐year follow‐up, the area of callus filling in the most medial side of the knee was measured using anteroposterior radiographs, and a modified van Hemert scoring system was used to evaluate bone union outcomes in five mediolaterally divided zones. Western Ontario and McMaster Universities Osteoarthritis Index (WOMAC) scores and relevant complications were assessed. The correlations between the gap width and bone union scores were evaluated.

**Results:**

Patients in the autograft group demonstrated better bone union progression (*p* = 0.031) and higher bone union scores (*p* < 0.01) compared to patients in the allograft or no graft groups. There were no significant differences in terms of postoperative WOMAC scores and incidence of complications among the three groups. No discernible linear relationships between the width of the opening gap and the bone union score were found.

**Conclusion:**

For MOWHTOs with an average gap opening width of 12.1 mm, autografts resulted in superior bone union outcomes compared to allografts and no graft at 1 year postoperatively. However, no bone graft achieved similar outcomes to allografts, suggesting that routine use of allografts should not be recommended.

## Graphical abstract

Autologous iliac crest, allogenous freeze‐dried tibia plateau, and no bone graft was used for medial opening wedge high tibial osteotomy gap filling. At 1‐year follow‐up, autografts resulted in superior bone union outcomes compared to allografts and no graft. However, no bone graft achieved similar outcomes to allografts, suggesting that routine use of allografts is not recommended.

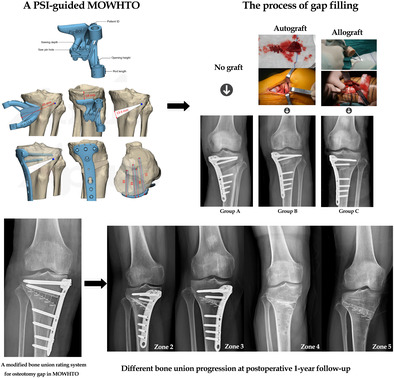

## Introduction

Medial opening wedge high tibial osteotomy (MOWHTO) has the potential to alleviate pain, improve function, and modestly modulate the natural history of knee joint degeneration by inserting a medial‐based wedge into the tibia to obtain valgus alignment and transferring the weight bearing line (WBL) away from the overburdened medial compartment.^1^, ^2^

The utilization of bone grafts or bone substitutes to fill the void caused by MOWHTO in the proximal tibia is a subject of ongoing debate. Furthermore, there remains uncertainty regarding the most suitable selection of a bone‐void filler from a range of available materials.^3^, ^4^, ^5^, ^6^, ^7^ There is a general consensus that autograft should be considered the “gold standard” for bone regeneration procedures.^8^ Autografts have exceptional osteo‐conductive and osteo‐inductive properties and offer strong structural support. However, the negative aspects of autograft can include limited sources, donor‐site morbidity, pain, prolonged operation time, and increased blood loss.^9^ The use of allograft could effectively address these drawbacks. Due to the rise in bone donation and the establishment of bone banks, accessing allograft has become more convenient.^9^ However, allograft comes with its own set of concerns, including a higher rate of graft resorption, diminished bioactivity and strength caused by processing, and poor integration with native bone.^10^

The existing research on autograft and allograft fillers has yielded conflicting findings regarding their clinical outcomes on bone union and postoperative function.^11^, ^12^, ^13^, ^14^, ^15^, ^16^ These results might have been confounded by factors such as variations in the size of the opening gap, diversities of surgical and fixation techniques, discrepancies in the duration of follow‐up, and demographic characteristics. In a meta‐analysis of randomized trials, Bei et al.^7^ found that bone grafting does not offer any benefits for osteotomy gaps smaller than 10 mm. However, they did note potential benefits for gaps larger than 10 mm. Conversely, Slevin et al.^5^ found no advantages in terms of union rates and correction loss when using various bone void fillers, but they did observe higher union rates with autografts. Fucentese et al.^11^ supported the notion that autografts promote bone union, but they found no significant effect on functional outcomes. In contrast, Sasaki et al.^17^ reported that b‐tricalcium phosphate significantly enhances bone formation and recommended using synthetic bone void fillers. Therefore, there is currently no consensus on the necessity of bone grafting and the preferred choice of graft material, mostly due to the existing low certainty level of evidence in this field.^18^

Therefore, the objectives of this study were twofold: (i) to compare the outcomes of bone union, patient‐reported scores, and the incidence of complications in patients undergoing MOWHTO with an opening gap larger than 10 mm using autograft, allograft, or no graft at 1 year postoperatively; and (ii) to determine whether the width of the opening gap is associated with differences in mean bone union score. The hypotheses of the present study were (i) autografts would lead to better bone union outcomes but would not significantly improve functional outcomes or reduce the incidence of complications; and (ii) there would be a negative correlation between the width of the opening gap and the bone union score.

## Materials and Methods

This was an institutional review board‐approved study of medical records from Sports Medicine Center of West China Hospital, and all participants gave valid informed consent to participate. This study was approved by the Health Sciences Research Ethics Board at Sichuan University and the local research ethics board (ID: WCH2018534).

### Patient Selection

**FIGURE 1:**
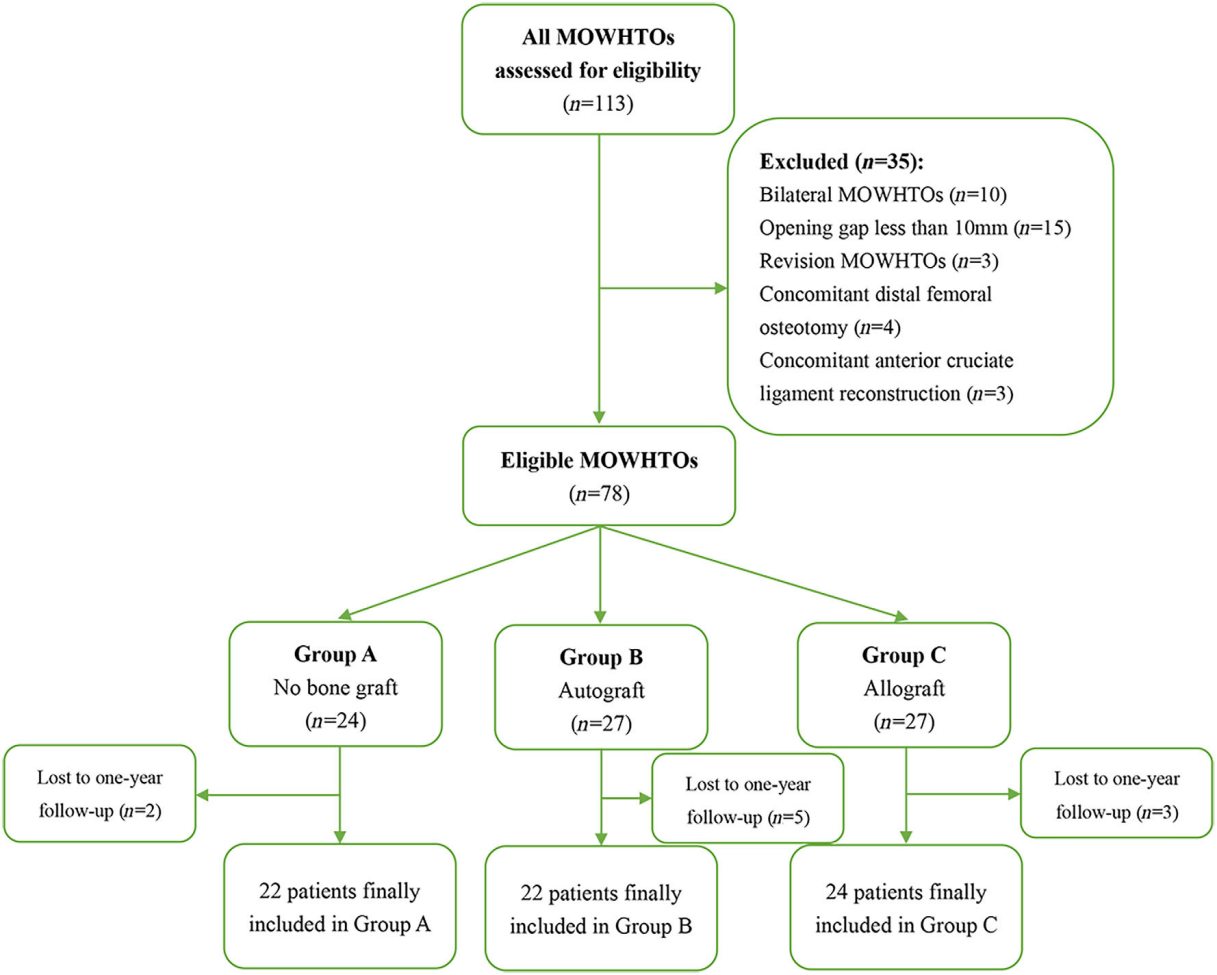
The flow diagram of patient enrollment. MOWHTO, medial opening wedge high tibial osteotomy.

Patients who were selected for the MOWHTO procedure exhibited symptoms and indications of knee joint osteoarthritis. These patients had previously undergone conservative treatments such as anti‐inflammatory medication, physical therapy, and activity modification, but did not experience satisfactory results. We retrospectively reviewed the medical records of all MOWHTO patients from January 2019 to December 2021. The inclusion criteria were: (i) patients aged 18 years or older with confirmed varus malalignment of the knee and mild to moderate osteoarthritis (Kellgren‐Lawrence grade I–III) in the medial compartment; and (ii) unilateral MOWHTO with an opening gap larger than 10 mm. Patients who met any of the following conditions were excluded from the study: bilateral MOWHTO, concomitant bony procedures, revision surgery, multicompartmental arthritis, a history of inflammatory arthritis, or inability to complete the 1‐year follow‐up. A total of 113 patients who had undergone primary MOWHTO were identified based on their medical records. After the screening process, the patients were divided into three groups according to the types of filler: no bone graft (Group A), iliac crest autograft (Group B), and freeze‐dried tibia plateau allograft (Group C), and 68 patients were deemed suitable for final inclusion in the study (Figure 1). The mean age of the patients at the time of surgery was 51.9 ± 6.1 years old, and the male:female ratio was 32:36. No significant differences were observed among the three study groups in terms of demographic data, as indicated in Table 1. Furthermore, there were no significant differences found among the three groups regarding the intraoperative correction angle and opening gap width. All patients were designated to undergo regular clinical assessments at 6 weeks, 3 months, 6 months, and 1 year after surgery. These assessments were conducted to monitor for any potential complications and evaluate any changes in rehabilitation protocols. However, specific requirements for radiographic examination and functional assessment were limited to the 1‐year follow‐up.

**Table 1:** Demographic and intraoperative data

Parameter	Group A (no graft)	Group B (autograft)	Group C (allograft)	*p*‐value
Male:female ratio	10:12	11:11	11:13	0.94†
Side (left/right)	11/11	10/12	10/14	0.85†
Mean age (range)	51.2 ± 5.4	54.2 ± 5.4	50.4 ± 6.9	0.09†
Height (cm)	160.2 ± 7.6	162.5 ± 6.3	159.2 ± 7.0	0.26*
Weight kg)	63.8 ± 6.4	66.5 ± 4.4	66.4 ± 4.9	0.23*
BMI (kg/m^2^)	26.1 ± 2.7	26.2 ± 3.1	26.8 ± 3.8	0.28*
Kellgren–Lawrence grade				
I	9	6	9	0.84†
II	10	11	12	
III	3	5	3	
Diabetes mellitus				
Yes	3	4	4	0.92‡
No	19	18	20	
Smoking				
Yes	2	1	2	0.83‡
No	20	21	22	
Correction degree (°)	10.9 ± 1.7	9.9 ± 3.0	11.2 ± 3.1	0.24*
Opening width (mm)	11.9 ± 2.3	11.4 ± 1.9	12.7 ± 2.9	0.44*

*Note*: The level of significance was set at *p* < 0.05.

Abbreviation: BMI, body mass index.

* One‐way analysis of variance.

† Pearson χ^2^‐test.

‡ Fisher exact test.

### Preoperative Planning and Surgical Technique

**FIGURE 2:**
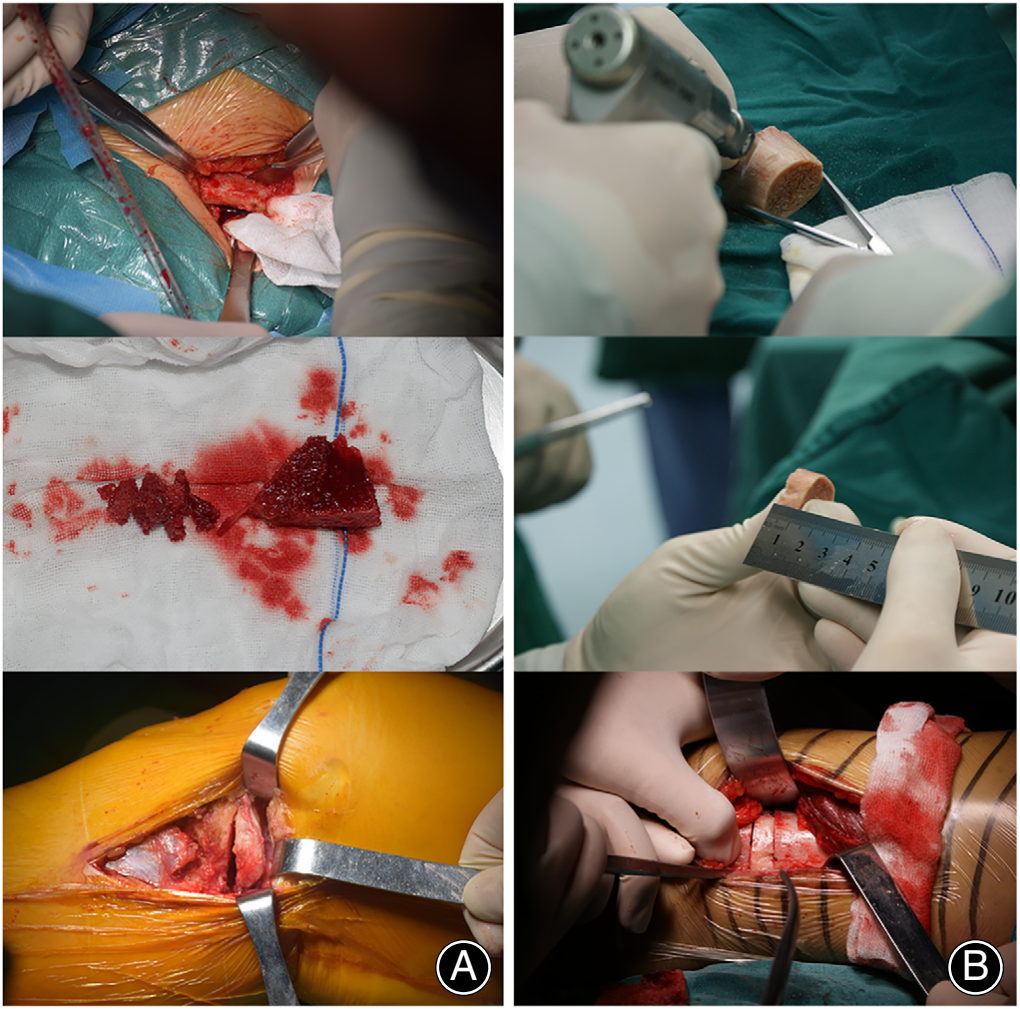
Use of autograft and allograft. (A) The harvest and implantation of iliac crest autograft in group B; at the bottom is a medial view of the right knee of a supine patient. (B) The preparation and implantation of freeze‐fried tibia plateau allograft in group C; at the bottom is the medial view of the right knee of a supine patient.

All HTOs were performed by a single senior doctor (J.L.). The preoperative planning strategy had been described elsewhere.^19^ Preoperative anteroposterior (AP) full‐length standing view and CT‐scanning images of the knee joint were used to determine the anatomical characteristics of the lower extremity, including the hip center, ankle center, joint line, mechanical axis, cutting point, and lateral hinge. The surgical parameters, including correction angle, WBL percentage, distraction angle, sawing direction and depth, were determined using Amira 4.0 software (Mercury Computer Systems, Berlin, Germany). The surgical technique of the biplanar HTO using a 3D‐printed patient‐specific instrument (PSI) had been described by Mao et al.,^20^ and this technique was applied to all included patients in this study. After the PSI‐based osteotomy, the osteotomy site was distracted according to preoperative planning, and fixation was achieved using a long locking compression plate system (Aplus, PSI Guider, Taiwan, China). For management of the osteotomy gap, autograft from the ipsilateral iliac crest (group B) (Figure 2A) or sterilized freeze‐dried tibia plateau allograft (group C) (Figure 2B) was used, while in some cases, the osteotomy gap remained unfilled (group A). The tibia plateau allograft in each case was provided by West China International Bone Bank (Chengdu, China). The allografts were prepared by removing the sterile packaging and placing the freeze‐dried grafts in sterile saline at room temperature for a duration of 15 min.

Initially, between January 2019 and January 2020, no bone graft or substitutes were used to fill the osteotomy gap. However, with the establishment of the West China International Bone Bank, our medical center transitioned to using either autograft or allograft. It is important to note that the decision on which type of graft to use was not randomized but instead based on the surgeon's judgment and the patient's input. Patients’ awareness and consent regarding the use of allograft, autograft, or leaving the gap unfilled were taken into consideration during this decision‐making process. There were no absolute criteria or indications that determined the choice of one graft type over the other in any of the cases.

### Radiological Assessment

Radiographic evaluations were performed using the AP full‐length standing view of the lower extremity, AP, and lateral radiographs of the knees taken preoperatively, immediate postoperatively, and at 1 year after the operation. The AP full‐length standing view of the lower extremity was standardized with the knee in full extension and with the patella facing forward at shoulder width in a weight‐bearing stance.

**FIGURE 3:**
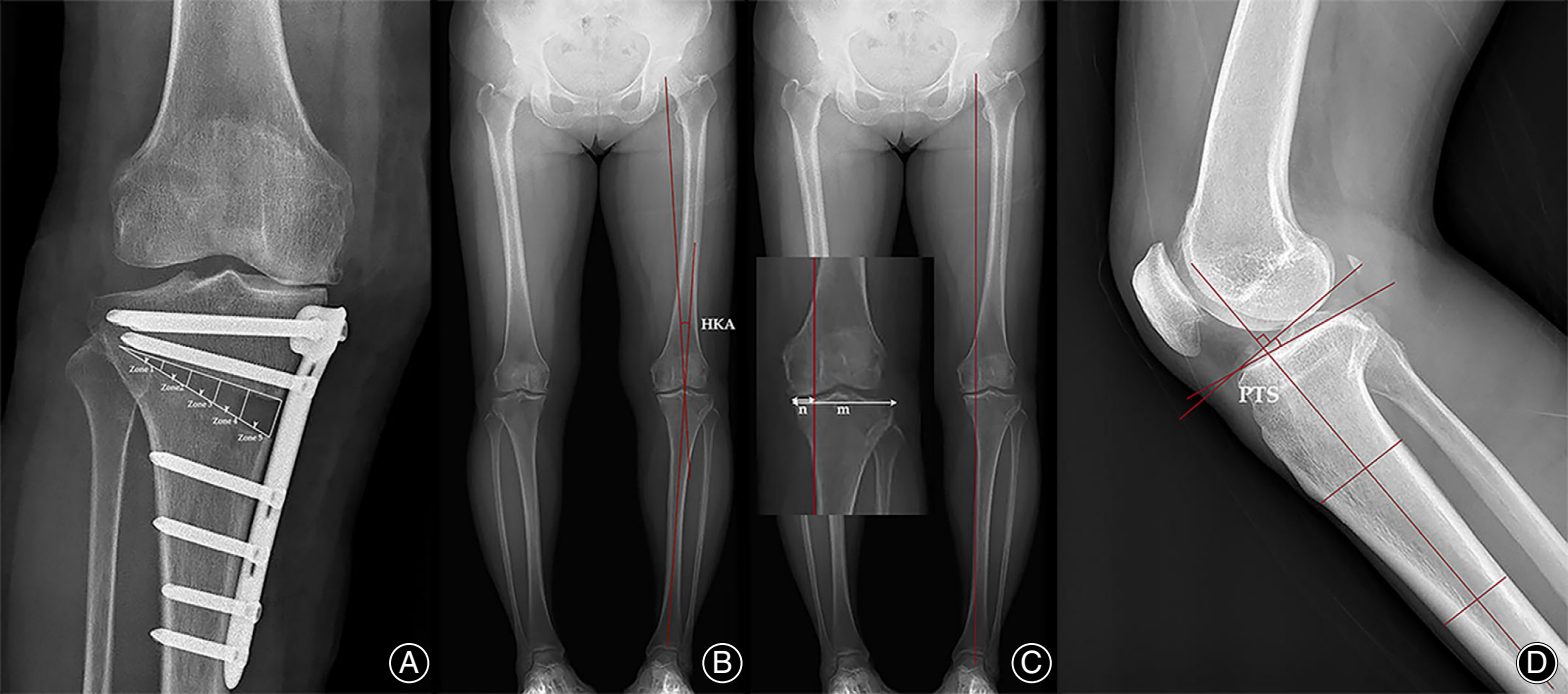
Radiographs illustrating the parameters that were evaluated. (A) The anteroposterior (AP) radiograph of a right knee showed a triangle consisting of two osteotomy lines originating from the opening gap and the medial cortex. This triangle was divided into five zones with equal base lengths from the lateral side. (B) The hip–knee–ankle angle was obtained with standing AP views of the lower limb by drawing a line from the center of the femoral head to the midpoint of the tibial spine and another line from this midpoint to the center of the talus surface of the ankle joint. (C) The weight bearing line (WBL) was drawn from the center of the femoral head to the center of the superior articular surface of the talus, the WBL ratio was calculated by dividing the distance from the medial edge of proximal tibia to the WBL (n) by the entire width of the tibia (m); (D) measurement of the posterior tibia slope. The proximal anatomic axis of the tibia is first drawn by connecting the mid‐cortical diameters of the tibia 5 and 15 cm distal to the joint line. A reference line is created perpendicular to this anatomic axis. The tibial slope is defined as the angle between the reference line and a line drawn tangent to the uppermost anterior and posterior edges of the medial tibial plateau.

Evaluation of the bone union pattern of the opening gap was performed by drawing a digitalized triangle along the borders and corner of the osteotomy on AP views, with five zones divided corresponding to 20% of the osteotomy along the base length from the lateral side (Figure 3A).^21^, ^22^ To assess the progress of bone union at the opening gap, the callus filling area on the most medial side of an anteroposterior radiograph was measured. The progression of bone union was tracked from the most lateral side to the most medial side. A minimum of 50% callus filling indicated stability in the osteotomy site.^22^ The modified van Hemert scoring grading system was used in each zone to assess the degree of bone union (Table 2). The bone union score was calculated by summing up the scores from each zone, providing an overall assessment of the bone union process.^13^, ^23^

**Table 2:** Modified van Hemert scoring system

Point	Explanation
a. With bone‐void filler (autograft of allograft)	
0	There was a clear distinction between the bone‐void filler and the surrounding bone on either the proximal or distal surface of the osteotomy gap
1	The osteotomy lines were not clearly visible, but there was a distinct, visible lucent line present on both the proximal and distal osteotomy surfaces
2	A blurred lucent line could be observed on a limited portion of both the proximal and distal osteotomy surfaces
3	A blurred lucent line was clearly visible on one osteotomy surface but not visible at all on the other surfaces
4	A blurred lucent line was visible on a limited part of one osteotomy surface but not visible at all on the other surfaces
5	No lucent line was visible on either of the osteotomy surfaces
b. Without bone‐void filler	
0	The surfaces of the osteotomy gap were clearly differentiated from each other
1	Although the exact location of the osteotomy lines became unclear, it was observed that the osteotomy gap did not show any signs of filling
2	Both osteotomy surfaces exhibited a blurred, translucent line, and the osteotomy gap was only partially filled without the presence of bridging callus
3	The bridging callus was visible on a limited part of the zone
4	The bridging callus was prominently visible, covering more than 50% of the zone, indicating a significant level of healing and bone formation
5	The osteotomy gap was totally filled

Other radiologic parameters including the hip–knee–ankle (HKA) angle (Figure 3B), the weight‐bearing line (WBL) ratio (Figure 3C), and the posterior tibia slope (PTS) were evaluated (Figure 3D). The PTS was defined as the angle between the medial tibial plateau and the anatomical axis of the tibial shaft on a lateral knee radiograph.

All radiological assessments were performed by two independent assessors (Yunhe Mao and Lei Yao) using ImageJ1 software (Rawak Software, Germany), and the two assessors (Y.M. and L.Y.) were blinded to the group allocation. Any disagreements between the assessors were resolved through discussion, with involvement from a third senior doctor (Y.X.) who did not participate in the surgeries, until a consensus was reached.

### Clinical Assessment and Complications

The clinical outcomes of the patients were assessed both before the surgery and during subsequent follow‐up evaluations using the Western Ontario and McMaster Universities Osteoarthritis Index (WOMAC) scores. The WOMAC scores provided a measure of the patients’ functional status (range, 0–68) and degree of pain (range, 0–20) and stiffness (range, 0–8).^24^

Various complications that occurred at any stage during the follow‐up period were carefully documented. Intraoperative complications included lateral cortical breach, which was characterized by radiographically visible disruption of the lateral proximal tibia. This breach could occur due to the extension of the osteotomy line into the proximal tibiofibular joint or propagation distal to that joint. Additionally, iatrogenic neurovascular injury was recorded as an intraoperative complication.

Postoperative complications that were taken into consideration included deep and superficial infections, wound dehiscence, deep vein thrombosis, pain syndrome, range of motion deficit, delayed bone union, loss of correction (LOC), as well as the need for revision or reoperation. Pain syndrome was defined as the presence of prolonged pain and/or pain that exceeded the normal postoperative levels. Delayed bone union was determined based on the extent of healing progress at the osteotomy site after 1 year postoperatively. Specifically, if the gap healing did not exceed 50% callus filling, it was categorized as a case of delayed union.^25^ LOC was defined as a difference of more than 3° in the HKA angle between the immediate postoperative and final follow‐up radiographs, indicating a possible loss of correction in the alignment of the lower extremity.^26^ Revision and reoperation were recorded as irrigation and debridement, as well as revision fixation.

Two skilled orthopaedic surgeons (J.L. and Y.X.) conducted thorough clinical assessments. They were unaware of the group assignments, and the documentation was meticulously recorded by a secretary. Any complications that arose were promptly addressed and managed.

### Statistical Analysis

The participants were retrospectively enrolled from a consecutive cohort, starting from the initiation of MOWHTO procedures at our medical center. The databases of observational study are usually used to answer casual questions.^27^ Thus, the estimated effect size of this study was not calculated. A comparison of continuous variables among the three groups was made with use of one‐way analysis of variance (ANOVA) and Kruskal–Wallis tests; the Pearson χ^2^‐test and Fisher exact test were used for categorical variables. Pearson correlation analysis was performed to evaluate the correlations between the widths of the opening gap and bone union scores in each study group. The intraclass correlation coefficient (ICC) was used to determine the interobserver reliability of all radiographic assessments. Continuous variables were presented as the means ± standard deviations (with range) and were analyzed using Stata (version 16.0). The level of significance was set at *p* < 0.05.

## Results

### Radiological Outcomes

**FIGURE 4:**
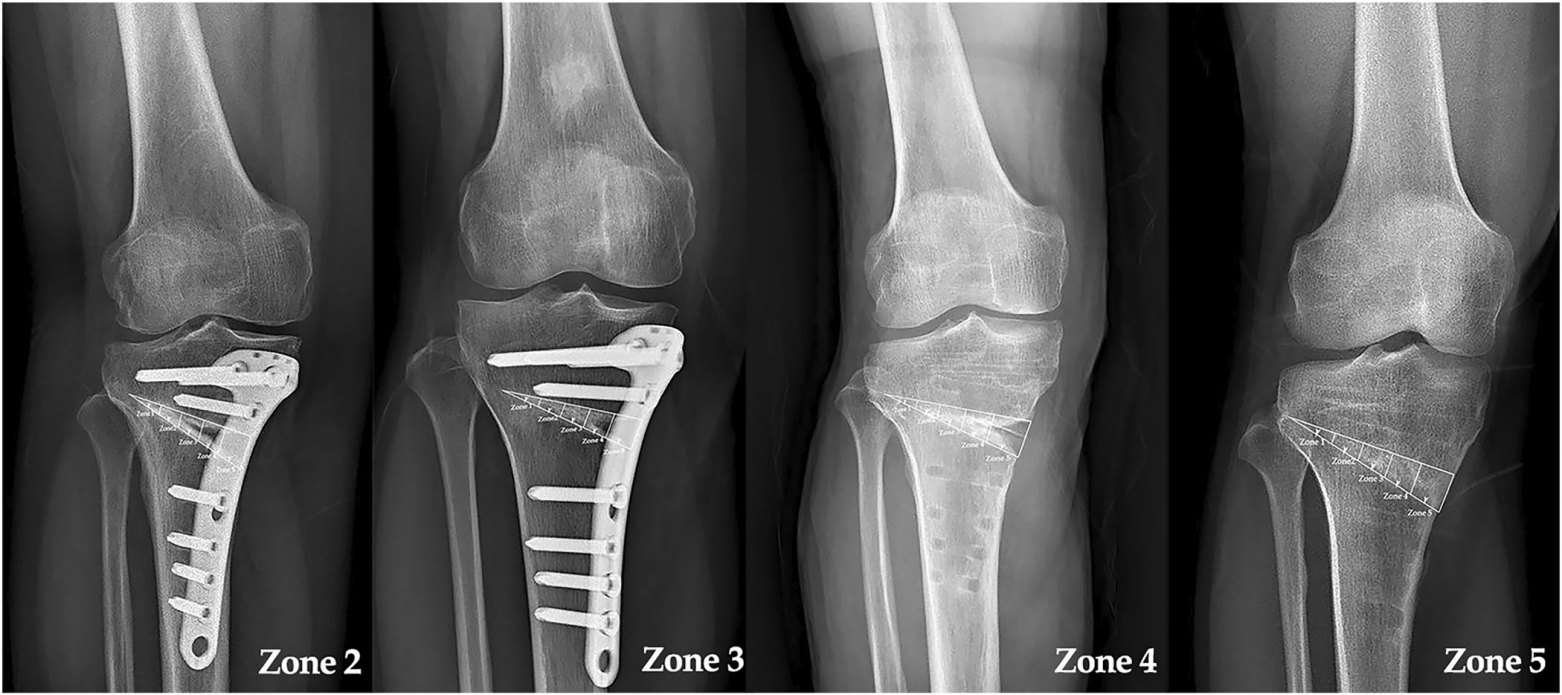
Different bone union zones at 1‐year follow‐up. Anteroposterior radiographs showing different bone union progressions from zone 2 to zone 5 at 1‐year follow‐up. From left to right, the gaps were filled with allograft, allograft, no graft, and autograft, respectively. When the gap was larger than 80%, filled hardware removal was considered.

The average interobserver ICC for the radiographic parameter measurements was found to be 0.87, with a range of 0.81–0.98. As indicated in Table 3, there were no significant differences among the three groups regarding the preoperative and postoperative values of the WBL ratio, HKA angle, PTS, and loss of correction degree. On analysis of the bone union progression based on the extent of mediolateral healing sites at 1‐year follow‐up, all knees in group B showed bone union through zone 4 or 5 (with van Hemert scores ≥ 4). However, 18.2% patients in group A and 29.1% patients in group C showed bone union through only zone 2 or 3, respectively. Further, the distribution of bone union zones is significantly different across the three study groups (Pearson χ^2^‐test, *p* = 0.031) (Table 3). Figure 4 shows different union patterns of the knees. In terms of bone union score, group B demonstrated a significantly higher score compared to group A (*p* = 0.017) and group C (*p* < 0.001). However, there was no significant difference detected between group A and group C (*p* = 0.09) (Table 3).

**Table 3:** Radiographic and clinical outcomes.

Radiological parameters	Group A (no graft)	Group B (autograft)	Group C (allograft)	*p*‐value	
Preoperative WBL ratio (%)	13.5 ± 13.4	10.9 ± 8.6	12.0 ± 10.3	0.73	
Postoperative WBL ratio (%)	61.7 ± 6.3	59.3 ± 8.4	58.4 ± 9.6	0.38	
Preoperative HKA angle (°)	170.5 ± 1.9	171.1 ± 2.3	171.0 ± 2.0	0.57	
Postoperative HKA angle (°)	181.4 ± 1.6	181.0 ± 2.3	182.1 ± 2.1	0.58	
Preoperative PTS (°)	9.0 ± 4.7	8.7 ± 5.1	8.6 ± 6.9	0.92	
Postop. PTS (°)	10.2 ± 4.3	9.8 ± 3.6	9.4 ± 6.2	0.97	
Loss of correction degree (°)	1.2 ± 1.3	0.8 ± 1.1	1.5 ± 1.5	0.15	
Bone union zone at 1‐year follow‐up					
Zone 5	10 (45.4%)	17 (77.3%)	9 (37.5%)	A versus B versus C	***p* = 0.031** *
Zone 4	8 (36.4%)	5 (22.7%)	8 (33.3%)	A versus B	***p* = 0.044** *
Zone 3	3 (9.1%)	0	4 (16.6%)	A versus C	*p* = 0.86
Zone 2	1 (4.5%)	0	3 (12.5%)	B versus C	***p* = 0.011** *
Zone 1	0	0	0	‐	
Bone union scores at 1 year follow‐up					
Total modified van Hemert score	19.1 ± 4.9	22.0 ± 2.3	17.8 ± 4.9	A versus B versus C	***p* < 0.001** *
				A versus B	***p* = 0.017** *
				A versus C	*p* = 0.09
				B versus C	***p* < 0.001** *
WOMAC scores					
Preop. total	44.2 ± 5.6	44.8 ± 7.1	46.2 ± 5.7	0.53	
Preop. pain	9.3 ± 1.0	10.1 ± 2.0	9.0 ± 1.4	0.11	
Preop. stiffness	4.4 ± 1.5	4.3 ± 1.4	4.3 ± 1.7	0.98	
Preop. function	30.5 ± 5.0	30.4 ± 5.3	32.9 ± 4.1	0.15	
Postop. total	25.2 ± 6.2	23.7 ± 5.6	26.8 ± 6.9	0.26	
Postop. pain	4.2 ± 2.1	3.9 ± 1.5	4.8 ± 1.6	0.26	
Postop. stiffness	4.0 ± 1.3	3.8 ± 1.3	4.2 ± 1.2	0.21	
Postop. function	16.9 ± 4.1	16.0 ± 4.2	17.8 ± 5.3	0.19	

*Note*: “Bone union zone” indicates the most medial zone with union with a van Hemert score of ≥4 (e.g., the value in Zone 5 represents the number and proportion with union in all 5 zones).

Abbreviations: HKA, hip‐knee‐ankle; Postop., postoperative; Preop., preoperative; PTS, posterior tibial slope; WBL, weight bearing line; WOMAC, Western Ontario and McMaster Universities Osteoarthritis Index.

* The level of significance was set at *p* < 0.05.

### Western Ontario and McMaster Universities Osteoarthritis Index Scores

Total WOMAC scores in three groups all improved at 1 year postoperatively, with no significant differences detected in the preoperative and postoperative values of pain, stiffness, and function scores among the three groups (Table 3).

### Complications

As for intraoperative complications, no popliteal artery injury nor peroneal nerve injury occurred in this cohort. Lateral cortex disruptions (lateral cortical breaches) were identified on instant postoperative radiographs, and the incidences were not significantly different among the three groups (*p* = 0.82) (Table 4). Most lateral cortical breaches (9/15) occurred in the earlier period of this study, which might have represented part of a technical learning curve. As for postoperative complications and revision or reoperations, no significant difference was detected among the three groups. There was one instance of superficial infection in group C, with persistent incision seepage and pain sustained after surgery; germiculture of the fluid implied an infection of staphylococcus epidermidis. Standard antibiotic therapy was applied, debridement and irrigation of the surgical site were performed. The wound seepage and pain disappeared thereafter, and the blood routine and pro calcitonin returned to normal. Thus, superficial infection was considered, and the hardware was retained.

**Table 4:** Incidence rates of complications.

Complications	Group A (no graft)	Group B (autograft)	Group C (allograft)	*p‐*value
Intraoperative complications				
Lateral cortical breach	4 (18.2%)	6 (27.3%)	5 (20.8%)	0.82
Popliteal artery injury	0	0	0	‐
Peroneal nerve injury	0	0	0	‐
Postoperative complications				
Superficial infections	0	0	1 (4.2%)	0.39
Deep infections	0	0	0	‐
Wound dehiscence	1 (4.5%)	0	1 (4.2%)	0.61
Deep vein thrombosis	1 (4.5%)	2 (9.1%)	1 (4.2%)	0.74
Pain syndrome	0	0	1 (4.2%)	0.39
Range of motion deficit	0	1 (4.5%)	0	0.35
Delayed bone union (callus filling <50%)	3 (13.6%)	0	5 (20.8%)	0.09
Loss of correction (>3°)	2 (9.1%)	1 (4.5%)	5 (20.8%)	0.26
Revision/Reoperation				
Revision fixation	0	0	0	‐
Irrigation and debridement	0	0	1 (4.2%)	0.39

*Note*: Lateral cortical breach: radiographically visible cortical disruption of the lateral proximal tibia (extension of the osteotomy line within the proximal tibiofibular joint; propagation distal to the proximal tibiofibular joint); Pain syndrome: having pain for a prolonged period and/or to a higher degree than normal postoperative pain; Loss of correction: loss of correction >3° between immediate postoperative and final follow‐up radiographs.

### Pearson Correlation Analysis

**FIGURE 5:**
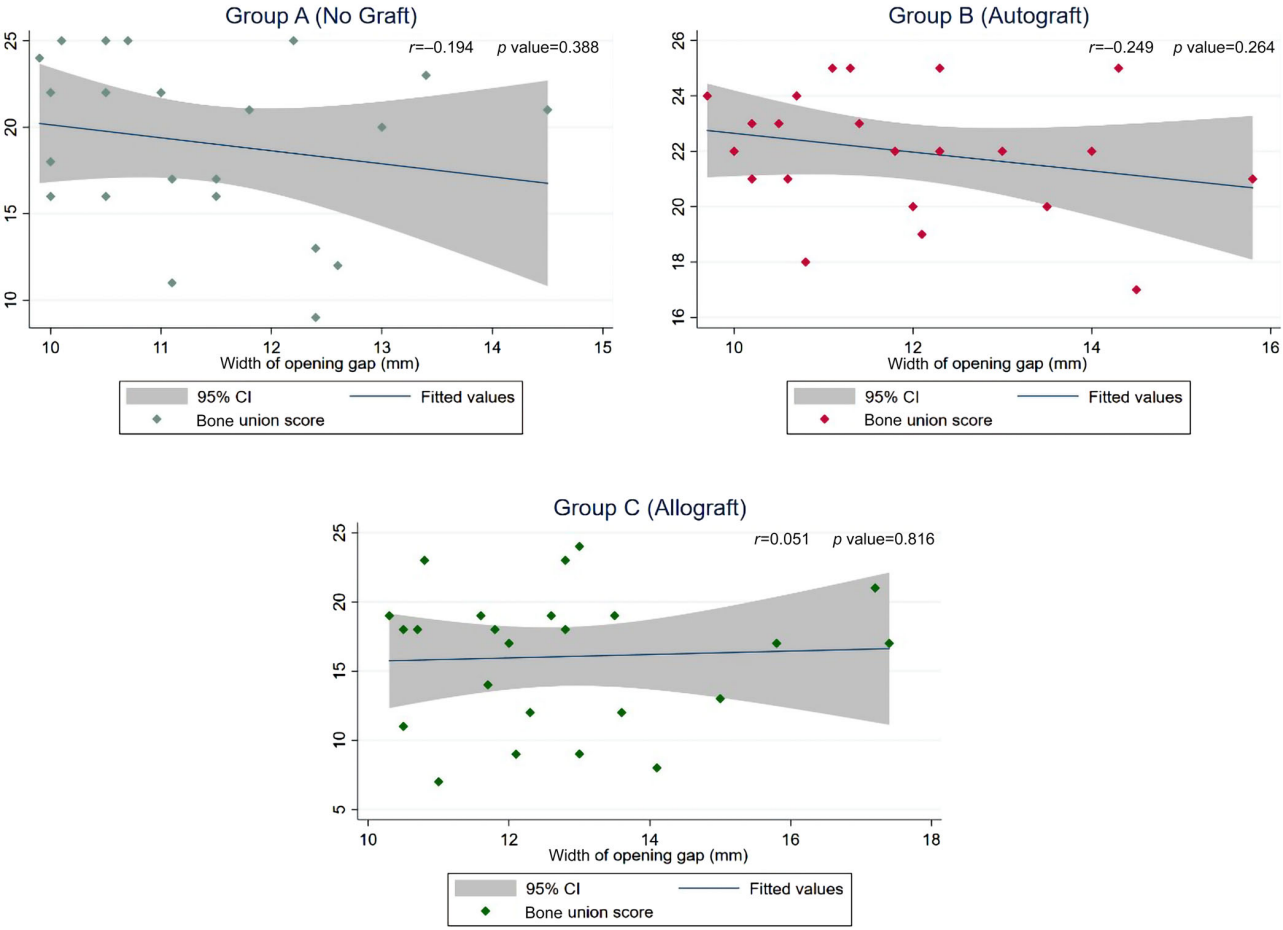
Pearson correlation analysis between gap width and bone union score. The scatter plots showed no discernible linear relationships between the width of the opening gap and the bone union score. The Pearson correlation coefficients (*r*) and *p*‐values were not statistically significant in all groups.

The scatter plots in Figure 5 revealed that there were no discernible linear relationships between the width of the opening gap and the bone union score in any of the three groups. This was further supported by the results of Pearson correlation analysis, which showed no significant correlation between the width of the opening gap and the bone union score in Group A (*r* = −0.194, *p* = 0.388), Group B (*r* = −0.249, *p* = 0.264), or Group C (*r* = 0.051, *p* = 0.816).

## Discussion

The principal findings of this study were: in MOWHTOs with opening gap ≥10 mm, (i) patients in the autograft group demonstrated superior bone union progression and higher bone union scores compared to patients in the allograft or no graft groups after 1 year. However, there were no significant differences in terms of postoperative WOMAC scores and incidence of complications among the three groups; (ii) there were no significant difference found between allograft group and no graft group in terms of postoperative bone union outcomes, WOMAC scores, and incidence of complications; and (iii) there were no correlations identified in opening gap width with the bone union score in all three groups.

### Radiological Outcomes

As for radiological outcomes of bone union, the autograft group exhibited a significantly higher bone union score as revealed by the ANOVA test (*p* < 0.01), indicating more complete bone healing compared to other groups. Additionally, the autograft group had more advanced bone union zones based on the Pearson χ^2^‐test (*p* = 0.031), suggesting a more favorable progression of bone healing at postoperative 1 year.

### Clinical Outcomes

No postoperative complications related to the donor site were observed, except for mild pain that did not require any intervention. There were no instances of hematoma formation or infection at the donor site. These findings highlight the positive outcomes and safety associated with the use of autografts in this study. However, it is important to note that no significant advantage was found for autografts in terms of WOMAC scores. According to a study conducted by Kim et al., patients undergoing MOWHTO need to achieve a 16.1‐point improvement in the WOMAC total score for it to be considered a minimal clinically important difference (MCID).^28^ In this current study, all three groups achieved this MCID from the preoperative to postoperative period without significant difference (*p* = 0.26), indicating a similar level of improvement in terms of functional outcomes regardless of the graft types. To determine the appropriate treatment, it is important to consider the effectiveness of the treatment as well as the individual needs of the patient. Currently, it is too radical to conclude that all gaps larger than 10 mm require autograft. Yet, in cases where the patient requires a faster and stronger bone union and expresses a preference for early removal of fixation, utilization of autograft would be the preferred choice.

### Pros and Cons of Allograft Filler

The use of an allograft gap filler has gained popularity owing to its potential in reducing perioperative pain and accelerating bone union by conferring structural support in MOWHTO.^29^ However, in this study, the use of freeze‐dried tibia plateau allograft as a filler in the osteotomy gap was ineffective in improving bone union outcomes. The group that did not receive bone graft achieved similar radiological and clinical outcomes compared to the allograft group at the 1‐year follow‐up. This might be explained by the fact that the process of freeze drying significantly diminishes the structural and biomechanical characteristics of the allograft.^30^ Immunogenic response has also been reported as a concern with freeze‐dried allografts, as this process may preserve the antigens in bone,^31^, ^32^ which could interfere with the natural healing of the bone and finally lead to resorption, nonunion, and recurrent pain.

Similarly, in a study by Kim et al., they found there was no difference between groups with allograft and without graft in terms of final bone union at 1 year postoperatively.^13^ Additionally, Kuremsky et al. reported a sixfold increase in the complication rate in the freeze‐dried allograft group compared to the autograft group.^33^ In contrast, the use of fresh‐frozen allograft yielded desirable bone union in MOWHTOs, and investigators were satisfied with the good clinical and radiographic results.^12^, ^29^, ^34^ Different donation sites of allografts (e.g., femoral head and iliac crest) and varied preservation methods of the allograft might shed some light on the different results. The discrepancy in quality of the allograft from a variety of suppliers should also be taken into account.

### Loss of Correction

Although the mean loss of correction angle was within an acceptable range (1.5 ± 1.5°), there was an incidence rate of up to 20.8% of loss of correction (>3°) observed in the allograft group at the 1‐year postoperative follow‐up. This incidence rate is significantly higher than the general average level of 6.9% (with a range of 0%–34.3%), as reported in a previous systematic review.^26^ It is uncommon for loss of correction to occur in locking plate high tibial osteotomy procedures.^35^ However, it is important to acknowledge that the difference in incidence rate can be attributed, in part, to the varying criteria used to define “loss of correction.” While the majority of studies consider a loss greater than 4° as loss of correction,^4^ we applied a narrower range of >3° in this study, resulting in an accelerated incidence rate. Additionally, the relatively small sample size might also introduce bias in the findings. In the current context, the safety and effectiveness of allografts in MOWHTO are unpredictable and dependent on various factors such as donation site, preservation method, and graft quality. Due to this uncertainty, the utilization of allografts may not be deemed superior to having no graft at all. As a result, it is difficult to make a recommendation regarding the routine use of allografts to fill the osteotomy gap in MOWHTO.

### Impact of Gap Size on Bone Healing

The width of the opening gap is indeed another important factor to consider in the analysis of gap healing following MOWHTO. According to previous other studies, bone grafting might not be necessary for small opening gaps (<10 mm) in MOWHTO.^5^

Goshima et al. studied the risk factors for delayed bone healing in MOWHTO and found that an opening width of more than 13 mm without bone fillers would lead to a delay in bone formation.^36^ In a study conducted by Dornacher et al., it was found that an opening wedge angle of 9° served as a threshold for the occurrence of bone healing disturbances.^37^ In this study, we included patients with opening gap ≥10 mm (range, 10.0–17.4), although the difference in delayed union incidence between the autograft group and the no autograft group was not statistically significant (Pearson χ^2^‐test, *p* = 0.07). It is important to note that this could be influenced by the different definition of “delayed union” used compared to other studies (e.g., considering osseous union if the site was ≥60% filled) and the relatively small sample size. If a stricter criterion for delayed bone union was applied or if the sample size was increased, there is a possibility that a significant difference could be identified. Furthermore, it is worth noting that the three cases of delayed bone union in group A had larger gap sizes than average, measuring 12.6, 13.4, and 14.1 mm. This might highlight the importance for surgeons to exercise caution when deciding on the appropriate filling method for larger gaps of over 12 mm.

### Strengths and Limitations

The strength of this study lies in its comprehensive investigation into the effects of different bone void fillers. It utilized a quantifiable bone union healing assessment system and conducted all‐sided radiological and clinical assessments, revealing factors that may affect gap healing. The study also had several limitations. First, confounding factors such as smoking, diabetes mellitus, and lateral hinge fractures were not accounted for, potentially affecting the accuracy of bone union results. This is because disruption of the lateral cortex can lead to excessive micromotion and hinder the healing process.^38^ Second, the study design was retrospective, meaning that data was collected from past records. The lack of randomization in group allocation introduces the possibility of selection bias, which could affect the generalizability of the findings. Third, we evaluated bone union using simple radiographs. Bone union can be judged more accurately with additional imaging such as computed tomography (CT) scans. However, given the medical costs and radiation hazards, we did not bring CT into practice. Finally, the sample size was small, consisting of only 68 patients. This limited sample size might restrict the ability to perform accurate statistical comparisons, especially between subgroups with different wedge sizes. It is important to consider these limitations when interpreting the results and applying them to larger populations. Future studies with larger sample sizes, randomized designs, and comprehensive imaging techniques are needed to address these limitations and provide a more robust understanding of the topic.

## Conclusions

In the context of MOWHTOs with an average gap opening width of 12.1 mm (range: 10–17.4 mm), our findings indicate that autografts yielded superior bone union outcomes compared to both allografts and no grafts at 1 year after the surgery. However, no bone graft group achieved outcomes comparable to the allograft group in terms of bone union score, WOMAC scores, and the incidence of complications. The safety and effectiveness of allografts in MOWHTO are unpredictable and dependent on various factors, suggesting that the routine use of allografts might not be recommended.

## Author Contributions

Yunhe Mao and Lei Yao were involved in collecting and measuring clinical and radiological data for the study, as well as conducting patient follow‐ups. They also contributed to drafting the paper. Yunhe Mao and Junqiao Li contributed to the statistical analysis of the data. Jian Li performed the surgical procedures for the study. Yan Xiong made significant contributions to the conception and design of the research. Furthermore, Yan Xiong reviewed the paper for important intellectual content and provided final approval for the version to be published.

## Ethics Statement

All procedures performed in studies involving human participants were in accordance with the ethical standards of the institutional and/or national research committee and the 1964 Helsinki Declaration and its later amendments or comparable ethical standards. Institutional Review Board approval was granted by the Institutional Review Board of West China Hospital, Sichuan University (No. WCH20180534).

## Conflict of Interest Statement

All authors have no conflicts of interest to report.
